# Isotonic Beverage Pigmented with Water-Dispersible Emulsion from Astaxanthin Oleoresin

**DOI:** 10.3390/molecules25040841

**Published:** 2020-02-14

**Authors:** Pedro Cerezal Mezquita, Carolina Espinosa Álvarez, Jenifer Palma Ramírez, Waldo Bugueño Muñoz, Francisca Salinas Fuentes, María del Carmen Ruiz-Domínguez

**Affiliations:** Laboratorio de Microencapsulación de Compuestos Bioactivos (LAMICBA) del Departamento de Ciencias de los Alimentos y Nutrición, Facultad de Ciencias de la Salud (FACSA), Universidad de Antofagasta, Avda. Universidad de Antofagasta, P.O. Box #02800, 1240000 Antofagasta, Chile; pedro.cerezal@uantof.cl (P.C.M.); jenifer.palma@uantof.cl (J.P.R.); waldo.bugueno@uantof.cl (W.B.M.); francisca.salinas@uantof.cl (F.S.F.); maria.ruiz@uantof.cl (M.d.C.R.-D.)

**Keywords:** isotonic drinks, oleoresin astaxanthin, emulsion, stability, antioxidant

## Abstract

Astaxanthin is a powerful antioxidant, because it neutralizes free radicals and plays a vital role in the prevention of human diseases. The objective of this work was to develop an isotonic beverage (IB) of orange-red color, using an astaxanthin oleoresin emulsion (AOE) that is dispersible in water. This was carried out in order to simulate the color of commercial isotonic beverages (CIB) prepared from artificial pigments. The size of the AOE micelles ranged from 0.15 to 7.60 µm^2^. The color difference (ΔE) was similar for the samples exposed to dark as well as light conditions. The samples subjected to light stress showed pigment degradation after seven days, followed by a decrease in the concentration of astaxanthin; whereas, the samples exposed to dark conditions remained stable for seven days and then showed a decrease in the concentration of astaxanthin (this decrease ranged from 65% to 76% when compared to the initial content) after a period of 91 days. For the astaxanthin oleoresin (AO) and AOE, the oxygen radical absorbance capacity (ORAC) values reached 5224 and 1968 µmol of trolox equivalents (TE)/100 g, respectively. When exposed to light conditions, the addition of AOE in the IB led to its rapid degradation, while it remained stable in the samples exposed to the dark conditions.

## 1. Introduction

Carotenoids are a group of molecules which consist of more than 750 pigments described. This group can be divided into carotene or xanthophyll subfamilies. Carotenoids molecules have been known for two features. Firstly, their color in solution varies between pale yellow, yellow, orange or red (ζ-carotene, xanthophyll, β-carotene or lycopene, respectively), and secondly, the positive link between higher dietary intake and tissue concentration of carotenoids plays a beneficial role in the human body due to the antioxidant properties that the group possesses, lowering the risk of occurrences of several degenerative disorders, such as types of cancer, cardiovascular or ophthalmological diseases [1,2].

Due to carotenoids color and beneficial health effects, these are widely applied in aqueous and oily matrices as colorants and additives. Nevertheless, carotenoids can degrade during food processing steps, like freezing, boiling or canning. The most important applications include pigmenting products such as margarine, butter, bakery products, sugar confectionery, meat, pasta and egg products, deserts and mixes, dairy and related products, fruit juices and beverages, canned soups, preserves and syrups [1,2].

**Figure 1:**
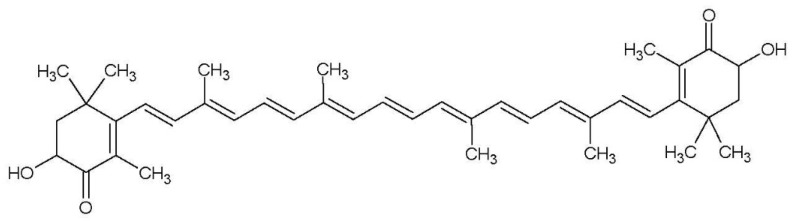
Chemical structure of bioactive compound astaxanthin.

The carotenoid “astaxanthin” belongs to a xanthophyll subfamily; in nature, this compound can be found in some green algae (like *Haematococcus pluvialis*), some fungi, bacteria and also occurs in some red algae by accumulation or synthesis [1]. The bioactive compound astaxanthin is characterized as a more effective antioxidant than vitamin C; it is 10 times more potent than β-carotene, lutein, zeaxanthin, canthaxanthin and 500 times more potent than α-tocopherol; therefore, it has been considered as a powerful antioxidant [3,4]. The presence of the hydroxyl and keto endings on each ionone ring (Figure 1) explains some unique features, such as the ability to be esterified, a higher anti-oxidant activity and a more polar configuration than other carotenoids [5,6]. Furthermore, astaxanthin has been used in diverse industries as a source of pigmentation. It received approval of the FDA in 1987 [7], and its daily consumption was established at 4 mg [4,5,6,7,8]. Nevertheless, it should be noted that astaxanthin cannot be completely absorbed by the human body because of its poor bioavailability. The low bioavailability of these kinds of functional lipids is because of their poor water solubility [9]. Hence, emulsions are required to dissolve the oil phase of astaxanthin in aqueous food matrices [10]. However, it should be considered that emulsions can lead to rupturing because of their composition; environmental conditions (temperature, pH, salinity, heat and freezing, among others) and processing conditions [11,12,13].

The isotonic beverages (IB) in the United States belong to the group of “sugar-sweetened beverages” and have been linked to weight gain in children and adults [14]. Over a period of time, their consumption has increased worldwide [15]. The formulation of IB constitutes carbohydrates (glucose, fructose and sucrose); electrolytes (Na^+,^ Cl^−^, K^+^ and others); occasionally complex B vitamin [16,17]; artificial coloring agents such as tartrazine, Allura red, sunset yellow, brilliant blue, etc. and has an approximate pH of 3.0 [16].

Some studies have reported the addition of astaxanthin and carotenes in emulsion form to beverages of the “instant-drink” and “ready-to-drink” types because of their polarity [18,19]. However, in the isotonic beverage form, the extracts of maqui, aҫai and berries have been reported to replace the artificial dyes and add the antioxidant property of anthocyanin [20,21]; similarly, there are reports of beverages pigmented with β-carotene [22]. Nevertheless, there were no previous reports citing the use of astaxanthin for pigmentation of isotonic beverages in the scientific literature.

The objective of this study was to develop an emulsion from the oleoresin of astaxanthin (AOE) obtained by supercritical extraction with CO_2_ and its application in IB to simulate the orange-red color imparted by synthetic dyes (such as tartrazine and red Allura AC) and to achieve an efficient antioxidant action. In addition, the stability of the pigment inside the IB was monitored for a storage period of at least three months.

## 2. Results and Discussion

### 2.1. Determination of the Size of the Micelles of Astaxanthin Oleoresin Emulsion at 10%

**Figure 2:**
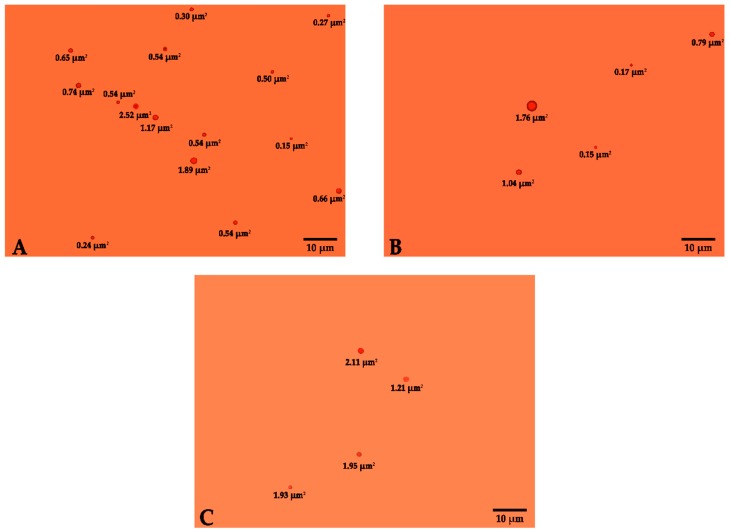
Micelles obtained in astaxanthin oleoresin emulsion at 10% in various dilutions in H_2_O (*v*/*v*) (**A**) 1:5, (**B**) 1:25 and (**C**) 1:50.

The emulsion was prepared as described in Materials and Methods; a homogeneous emulsion was visible to the naked eyes. For the classification of micelles, it was necessary to know their measurement and the emulsion quality. The characteristics of the generated micelles are shown in Figure 2, where the size obtained (areas) from micelles could be observed in the dilutions of 1:5, 1:25 and 1:50; in Figure 2B, the micelles ranged from 0.15 to 7.60 µm^2^ in the emulsion. This is classified as a macro-emulsion, according to [23], as the area ranged from 0.008 µm^2^ to 0.008 mm^2^ and its diameters were in the range of 100 nm to 100 μm. With respect to emulsions, several types of stability problems can also occur, causing them to be thermodynamically unstable and affected by flocculation and coalescence phenomena, as described previously in other studies [24,25]. The incorporation of sucrose monoester (surfactant) in the emulsion helps its stabilization [26]. Nevertheless, the obtained micelle size is acceptable for the food industry, since it lies within the range of 0.03 µm^2^ to 0.008 mm^2^ [27]. 

### 2.2. Chromatic Characteristic in Commercial and Pigmented AOE with Isotonic Beverages 

Table 1 shows the chromatic coordinates obtained for the different commercial isotonic beverages. In the absence of any colorant, the isotonic beverage had a luminosity of $L^{*}$ = 61.72 ± 0.56, (colorless) indicating a slight tendency toward white color with respect to the $L^{*}$ scale (0 to 100). For the orange-colored commercial isotonic beverages (A and B), no significant differences (for *p* < 0.05) were obtained for the coordinates $L^{*}$ and $b^{*}$, unlike those obtained for the coordinate $a^{*}$. However, for the red-colored commercial isotonic beverages of brands A and B, significant differences (*p* < 0.05) were found in all the three chromatic coordinates: $L^{*}$, $a^{*}$ and $b^{*}$. On the other hand, the index of redness in the orange-colored drinks was in the range of 0.3, while in the red-colored ones, they exceeded the value of 1.0, as expected.

**Figure 3:**
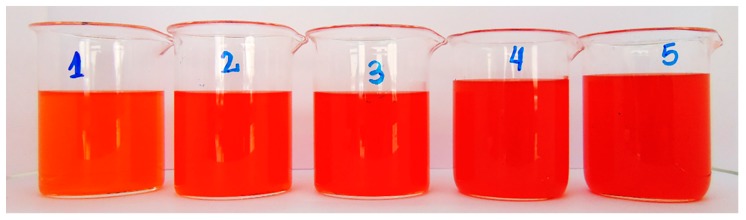
Photograph of isotonic beverages pigmented with the addition of astaxanthin oleoresin emulsion (AOE), where 1 and 5 are the lowest and highest concentrations, respectively.

Table 2 shows the chromatic coordinates obtained by each prototype of the pigmented isotonic beverages (PPIB) with statistically significant differences (*p* < 0.05). It was found that the values of the chromatic coordinate $L^{*}$ were inversely proportional to the concentration of added astaxanthin oleoresin emulsion, while the values of the chromatic coordinate $a^{*}$ proved to be directly proportional to the concentration of astaxanthin oleoresin emulsion; this trend was not observed for the chromatic coordinate $b^{*}$. Figure 3 shows the obtained color for all prototypes.

Table 3 presents the color differences (ΔE) between the mean values of chromatic coordinates of commercial isotonic beverages (Table 1) and mean values of pigmented IB (Table 2) during time = 0; that is, the $L^{*}$, $a^{*}$ and $b^{*}$ chromatic coordinates were measured as soon as the IB was pigmented with AOE, and then ΔE values were calculated. The ΔE values of the orange-colored commercial beverages of both brands (A and B) and the pigmented beverages developed with the incorporation of AOE ranged from 31.94 to 45.57. On the other hand, the ΔE values of the red-colored commercial beverages of both brands (A and B) and the pigmented ones developed with the incorporation of AOE had lower values between 8.05 and 19.96. However, there were significant differences (*p* < 0.05) between the concentrations of each of the brands (A and B) for the two colorations (orange and red), indicated by the first letters in the exponents of the values of each column, except between formulations 3 and 4 of brand A (red). Similar behavior was obtained upon a comparison of the values between A and B brands of both colors (orange and red), corresponding to the second letters in the exponents of the values of each row (Table 3).

From Table 3, we can see that the lowest value of ΔE corresponded to 8.05 ± 0.09 (formulation no. 3), which is the red commercial IB of brand A. This ΔE value is high enough to be perceived by the consumers, since it is indicated that the human eye can detect color difference if ∆E > 5.0, although some people with better vision can visualize the differences with a value of ∆E > 3.0 [28,29,30]. Based on this result, the commercial IB of brand A was selected for further analyses of stability based on color. Formulation 3 of pigmented IB was called the “prototype pigmented isotonic beverage” (PIBP).

### 2.3. Stability of Astaxanthin Concentration and Variation of Chromatic Coordinates in PIBP for a Period of Three Months

**Figure 4:**
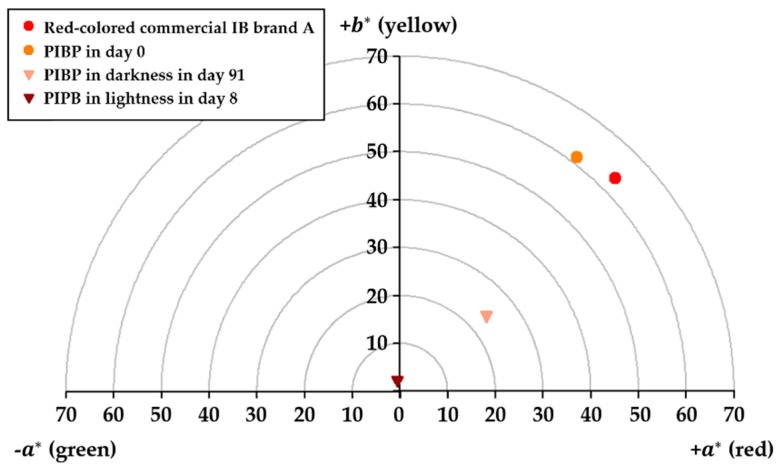
Top of the color sphere, quadrants first and second showing the locations of red-colored commercial isotonic beverages (IB) of brand A, and prototype pigmented isotonic beverages (PIBP) in its three conditions, at time 0, stored in light stress and stored in darkness.

Figure 4 shows the occupied positions of the commercial IB of brand A and the PIBP at different moments of its storage under light and darkness stress—in the upper two quadrants of the colored sphere. The commercial IB brand A presented the same values as shown in Table 1, where $L^{*}$ = 33.34 ± 0.17, $a^{*}$ = 45.09 ± 0.06 and $b^{*}$ = 44.40 ± 0.45. On the other hand, PIBP presented a value of $L^{*}$ = 32.29 ± 0.06 at time 0 and values of chromatic coordinates $a^{*}$ and $b^{*}$, being 37.05 ± 0.05 and 48.88 ± 0.21, respectively. On the other hand, PIBP exposed to light stress presented a value of $L^{*}$ = 64.59 ± 0.39; the values were −0.64 ± 0.10 for $a^{*}$ and 2.22 ± 0.16 for $b^{*}$, respectively. Storage of PIBP in the dark for three months presented an $L^{*}$ value of 48.51 ± 0.07, and the chromatic coordinates were $a^{*}$ = 18.03 ± 0.08 and $b^{*}$ = 15.80 ± 0.13, respectively. The ΔE for PIBP samples stored between zero and three months was 9.27 ± 0.13. The changes in color in PIBP stored under light stress could be explained by the process of photodegradation of carotenoids; this phenomenon has been reported in optically transparent systems, where the waves of light can pass more easily than in other optical systems [31]. Additionally, it can be established that AOE had a tendency to destabilize in the solution, hence affecting its concentration and getting reduced during storage. This condition is observed to a lesser extent at low temperatures; it gets aggravated by the unsaturated structure of astaxanthin and its sensitivity to heat, light and oxygen [32].

**Figure 5:**
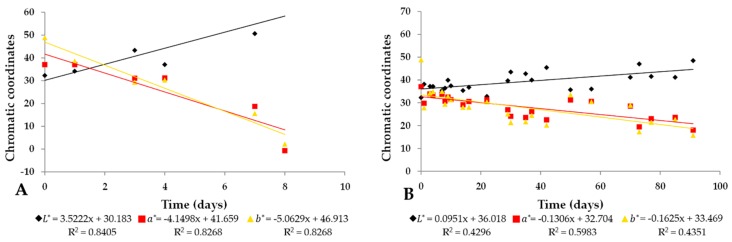
Variation in chromatic coordinates and qualitative characteristics over time. (**A**) PIBP storage under light stress and (**B**) PIBP storage in darkness. R^2^: linear behavior.

Figure 5 shows the variation of the chromatic coordinates and qualitative characteristics experienced by PIBP during a period of eight days under light stress and three months of storage in dark.

It was observed that the values of the chromatic coordinate $L^{*}$ of PIBP subjected to light stress (Figure 5A) doubled with respect to the initial values, ending in 64.59 ± 0.39 on day eight, showing a linear behavior of R^2^ = 0.8405, while the chromatic coordinates $a^{*}$ and $b^{*}$ showed a decrease of more than four times of their initial values, also with a linear behavior, reaching the mean values of $a^{*}$ and $b^{*}$ equal to −0.64 ± 0.10 and 2.22 ± 0.16, respectively, and with R^2^ = 0.8268 and R^2^ = 0.9501, respectively.

Figure 5B presents samples stored in dark for 90 days. Here, also, we could observe an increase in the $L^{*}$ coordinate but with less significance than the $L^{*}$ of samples recorded at the beginning of the storage period in the dark. It reaches a mean value of 48.51 ± 0.07 and has a low coefficient of determination of linear behavior (R^2^ = 0.4296). On the other hand, the chromatic coordinates $a^{*}$ and $b^{*}$ had final mean values of 18.03 ± 0.08 and 15.80 ± 0.13, respectively, and lower values of R^2^ of 0.5983 and 0.4351, respectively.

The tendency presented by IB in both conditions, light stress and dark, is shown in Figure 5A,B. The values of $L^{*}$ for IB exposed to light stress were similar to those observed in IB pigmented with anthocyanins obtained from the eggplant when stored at 4 °C and 25 °C and protected from light; it was also shown that the intensity of lightness $\left(L^{*}\right)$ increased with storage time (Arrazola et al., 2014). On the other hand, in emulsions containing β-carotene, it has been observed that the values of the coordinates $L^{*}$, $a^{*}$ and $b^{*}$ decrease over time [33]. However, it has been established that, in emulsions, the values of lightness ($L^{*}$) increase with time, whereas the values of $a^{*}$ and $b^{*}$ decrease [34]. It can be established that temperature was not a factor that affected the degradation of the samples subjected to light stress, since the carotenoids in apple juice show a decrease in lightness at temperatures above 60 °C [35].

When exposed to light stress, it is observed that carotenoids—especially the astaxanthin—promote the formation of isomers (cis-trans) by means of photoexcitation. This ruptures the double bonds, causing photodestruction of the molecule and the resultant loss of the pigment color [36,37,38]. This observation thus explains the time difference observed for degradation of the astaxanthin compound in the samples stored under light stress, compared to those stored in dark (greater degradation in samples stored in light stress).

### 2.4. Color Variation (ΔE) of Prototype Isotonic Beverage Pigmented (PIBP) over a Period of Time

**Figure 6:**
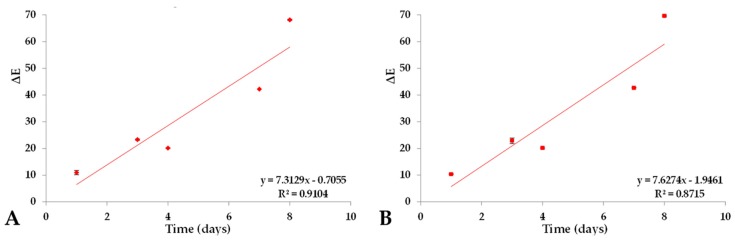
Color difference (ΔE) during the stability analysis. (**A**) is a comparison between PIBP in the day 0 and next days, under light stress storage condition. (**B**) is a comparison between PIBP under light stress condition, and red-colored commercial IB (brand A).

Figure 6 shows the color differences (ΔE) of PIBP under conditions of light stress and the ΔE experienced by PIBP in relation to red-colored IB of brand A over a period of eight days. The PIBP at time zero presented an almost seven-fold increase under conditions of light stress (Figure 6A), with an initial value of 10.44 and a standard deviation of 1.36 and a final value of 68.12 and deviation of 0.18. During the stability analysis, there was a seven-fold increase in the color variation between the red-colored IB of brand A and PIBP under light stress (Figure 6B) upon storage. The initial ∆E was found to be 9.27 ± 0.13, and the final value of 69.62 ± 0.15 was observed. The color differences of PIBP under light stress can be related to the degradation of astaxanthin through photoisomerization, since astaxanthin oxidizes rapidly. However, we must also take into account the fact that water activity, temperature, presence of oxidants or antioxidants and other elements also influence the involvement of a food product in the degradation of carotenoids [37,38].

**Figure 7:**
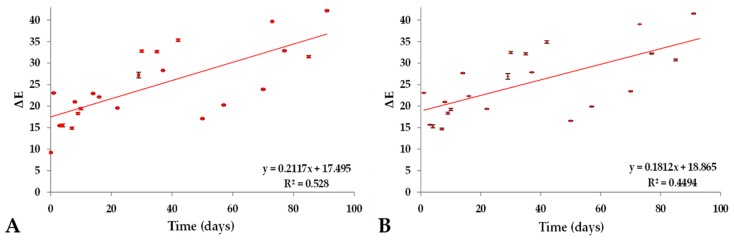
Color difference (ΔE) during the stability analysis. (**A**) is a comparison between PIBP under darkness storage condition and red-colored commercial IB (brand A). (**B**) is a comparison between PIBP day 0 and next days under darkness storage condition.

Figure 7 shows the color differences (ΔE) in PIBP under the dark condition and the ΔE experienced by PIBP in relation to red-colored IB of brand A over a period of three months. A 4.5-fold increase in the color difference (ΔE) between red-colored IB of brand A and PIBP in dark conditions (Figure 7A) was experienced compared to its initial value (9.27 ± 0.13); it had an ending value of 42.19 ± 0.11. For dark conditions (Figure 7B), the variation doubled with an initial ΔE value of 23.00 and a standard deviation of 0.23 and a final value of 41.46 with a standard deviation of 0.19. Therefore, the phenomenon of pigment decolorization could be established in both conditions, providing greater protection to the pigment when stored in the dark. The degradation in this condition may partly be the result of some kind of emulsion breakdown, considering the fact that it is a macro-emulsion and is thermodynamically unstable [39].

### 2.5. Stability of Astaxanthin Developed in the Isotonic Beverage

**Figure 8:**
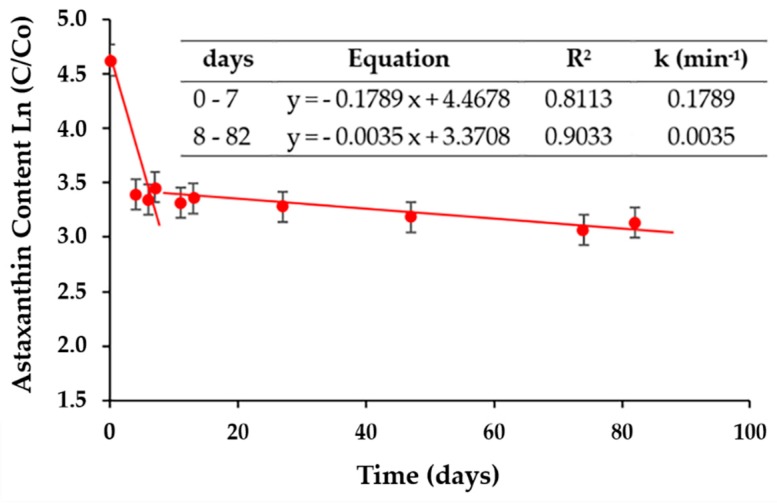
Degradation of astaxanthin in the isotonic beverage samples stored in darkness for three months.

Figure 8 shows the stability of astaxanthin in PIBP over a period of three months of storage under darkness, indicating a nonlinear relationship between ln(C/C0)∗100 and time. This nonlinear function is an expression of the behavior of astaxanthin oleoresin inside the beverages. Our observations are similar to the results of other authors, where it has been described that the linear degradation models are not applicable for the astaxanthin pigment after a few weeks of storage [40]. Additionally, [9] have shown that the loss of astaxanthin could obey a polynomial nonlinear function form in the preparation of the emulsion, indicating that there could be other factors like vitamin, sugar, mineral salts and others affecting the stability of astaxanthin in the beverages. However, two linear stages of nonlinear behaviors were assumed, one for the first seven days and another for the rest of the storage, with which the events can be empirically explained. For the first seven-day stage, a linear condition was observed with a value of R^2^ = 0.8113 with a degradation rate of k = 0.1789 min^−1^, while, for the second, after eight days, a value of R^2^ = 0.9033 and k = 0.0035 min^−1^ was obtained. This indicates that the degradation rate is 51.1 times higher in the first stage than in the last stage; in other words, the value of k in the second stage is 19.6% less than the value of k in the first stage.

It was stablished that, during the first week, the astaxanthin concentration decreased to 27.4%, this loss being attributed to the possible instability of the pigment within the matrix of the beverage. From that moment until the end of storage for three months, the astaxanthin concentration was between 64.59% and 75.72% of the initial astaxanthin concentration (*t* = 0 day). This indicates that the astaxanthin concentration in the isotonic beverage remains practically stable after the first week; being an important advantage of the product.

The behavior presented by astaxanthin as a part of the isotonic beverage was consistent with the results of other studies on isotonic beverages; those are pigmented with anthocyanins and β-carotene, where a slight decrease in the concentration of pigments was observed after storage in dark conditions and under low temperatures [22,41,42].

### 2.6. Analysis of Some Control Variables of the Prototype Isotonic Beverage Pigmented (PIBP)

Table 4 shows the values obtained for some control variables, soluble solids (SS) and pH indices during the stability analysis conducted for three months. The mean values of the SS of PIBP were maintained throughout the storage between 1.3 to 1.4 °Bx, and the differences were not statistically significant (*p* < 0.05). On the other hand, the mean values of pH for the samples stored in dark showed a slight increase at the end of the storage period, with statistically significant differences (*p* < 0.05). However, there was no significant difference between the start and the middle of the storage periods (*p* < 0.05). The astaxanthin pigment from *Phaffia rhodozyma* demonstrated higher stability at 40 °C and a pH of 4.0; the stability decreased with an increase in the pH [18,19,20,21,22,23,24,25,26,27,28,29,30,31,32,33,34,35,36,37,38,39,40,41,42,43].

Studies carried out on nano-emulsions of carotenoids, β-carotene and lycopene established that, at pH values ≤ 3.0, the color degraded within a period of five days. Moreover, pH values oscillating between 4.0 and 5.0 denoted instability in emulsions, due to an increase in the particle size and separation of the phases owing to the creaming phenomenon [33,34,44,45]. The concentration of chlorides in commercial colorless IB was 0.4 ± 0.2 g/100 mL; it remained stable when AOE was added to commercial colorless IB to impart the coloration, and no statistical differences (*p* < 0.05) were observed.

### 2.7. Analysis of Capacity Antioxidant Assay in Oleoresin Astaxanthin Emulsion (AOE)

The oxygen radical absorbance capacity (ORAC) assay value of AOE was 1968 µmol Trolox equivalents (TE)/100 g. According to the amount of AOE added to PIBP (2235 AOE/g PIBP), the ORAC value corresponding was 0.04398 µmol TE/g PIBP. This value, added to isotonic beverage without colorant of 0.20 µmol TE/g [46], was 0.244 µmol TE/g PIBP. When comparing this ORAC value with those reported in Table 5 for commercial isotonic beverages (1.13 ± 0.04 µmol TE/g) and yogurt (1.76 µmol TE/g), the obtained value for PIBP was below expectations; however, it was higher than the sweetener values (0.08 ± 0.02 µmol TE/g) [47].

Whereas, the ORAC value for 600 mL (2.5 portion of 240 mL each of PIBP) was 146.4 µmol TE/600 g PIBP; however, with this quantity of PIBP, the consumer can have an 8.13% of the daily consumption for an American person, according to the USDA [48].

The results achieved in the total polyphenol test for AOE was 78.00 mg gallic acid equivalents (GAE)/100g. According to the quantity of AOE added to PIBP (2.235 AOE/g PIBP), the corresponding value was 0.00174 mg GAE/g PIBP, which represents 0.174 mg GAE/100 g PIBP. This value, added to the isotonic beverage without colorant with 0.63 mg GAE/100 mL, was the maximum value obtained by [49], with 0.804 mg GAE/100 g of PIBP and being within the range reported by [49] for commercial isotonic beverages from 0.48 a 5.70 mg GAE/100 mL and below other noncommercial isotonic beverages with berries from 5.90 to 80.97 mg GAE/100 mL and other kind of berry juices ranging from 0.4 to 1.7 mg GAE/mL [50].

## 3. Materials and Methods

### 3.1. Materials

Isotonic beverages (IB) of two brands (A and B) were selected from the local market of Antofagasta City, choosing ones that specified the addition of yellow synthetic dyes, such as “sunset yellow” and “tartrazine” on their labels and others of red color that indicated the addition of red synthetic dye “red Allura AC” on their labels. All IB bottles, like major consumer plastic sports drink bottles, are made from PET material and contain a volume of 1 L. A colorless IB was also selected, which had no dye addition (brand A) (according to its label).

The astaxanthin oleoresin (AO) extracted at 10% from the *Haematococcus pluvialis* biomass by the supercritical CO_2_ extraction technology was supplied by Atacama Bio Natural Products Inc., Iquique, Chile.

Propylene glycol; sodium sulfate (both analysis quality) and the reactants: acetone, petroleum ether, *n*-hexane, dimethyl sulfoxide and water (high-performance liquid chromatography (HPLC) grade) were purchased from Merck. Grade 2 table sugar was obtained from the Jumbo Supermarket in Antofagasta City, Chile, and the surfactant (Habo Monoester P90) was provided by Compass Food (Singapore, Singapore).

### 3.2. Preparation of Astaxanthin Oleoresin Emulsion (AOE)

For the preparation of the AOE, the [51] method was used with some minor modifications. Liquid phase comprising of propylene glycol and water (1:2.7, *v*/*v*) was mixed with the solid phase comprising of sugar and Habo Monoester P90 (19.52:1, *w*/*w*) and heated at 45 ± 5 °C until the sugar dissolved completely. This mixture was then homogenized at high speed with a Bio-Gen PRO200 homogenizer (PRO Scientific, Oxford, CT, USA) at 35,000 rpm for 6 min; 6.29% of astaxanthin oleoresin at 10% concentration was added and stirred again for 5 min. Immediately, this was disrupted by sonication with an ultrasound probe (CP130 ultrasonic processor Cole-Parmer, Vernon Hills, IL, USA) at 60% of maximum amplitude (130 W, 20 kHz) at pulsed mode (6 s ON/6 s OFF) for 20 min. An increase in temperature was prevented by the use of an ice bath maintained outside the beaker. All these steps of homogenization helped reduce the size of micelles formed. Finally, the emulsion was packed in amber glass flask (10 mL) with a plastic screw cap, wrapped with a foil film and stored at 4 °C.

To determine the size of the AOE micelles, we prepared three dilutions such as 1:5, 1:25 and 1:50 (*v*/*v*), and their images were captured with a microscope (CX31, Olympus, Tokyo, Japan) and an integrated digital camera. The image-processing software Micrometrics SE Premium for Windows was used for analysis, which is a suitable and frequently used tool when other equipment for determining physical measures is not available. For this purpose, the surface area of the micelles was determined from three random zones of the observation field of each dilution selecting those areas where the micelles were larger in size. The area was determined using Equation 1:

(1) $$A=\;\pi *r^{2}$$

where A= area; π= Pi number and r= ratio of each micelle.

### 3.3. Chromatic Coordinates Measurement

Colorimeter (Colorflex 45°/0°, HunterLab, Reston, VA, USA) possessing the CIE $L^{*}a^{*}b^{*}$ system was used. The samples (25 mL) were taken, and 10 readings were recorded for each sample. The color difference in the samples (ΔE) was determined using Equation (2):

(2) $$\Delta E=\;\sqrt{\Delta L^{2}+\Delta a^{2}+\Delta b^{2}}$$

where

$\Delta L=L_{2}^{*}-L_{1}^{*}$ (luminosity difference),
$\Delta a=a_{2}^{*}-a_{1}^{*}$ (red-green difference) and
$\Delta b=b_{2}^{*}-b_{1}^{*}$ (blue-yellow difference).

The subscripts 1 and 2 indicate two moments of the measurement (initial and final times) between which the color difference was calculated.

### 3.4. Preparation of Pigmented Isotonic Beverage Prototype (PIBP)

Five independent aliquots of colorless commercial isotonic beverages were selected: 0.53, 0.99, 1.55, 2.08 and 2.52 mg/g. The color difference among these prototypes of the colored beverages was determined. To perform the stability analysis with a commercial isotonic beverage (which had the addition of artificial colorants), the prototype with the least color difference was chosen.

An isotonic beverage pigmented with AOE at 1.55 mg/mL was prepared for the stability analysis, and the mixture was homogenized by stirring with an ULTRA-TURRAX at 35,000 rpm. This formulated beverage was called the pigmented isotonic beverage prototype (PIBP).

### 3.5. Storage of Pigmented Isotonic Beverage Prototype (PIBP)

The PIBP was packaged at a room temperature of 14.3 ± 3 °C in sterile glass jars of 28 mL provided with twist-off caps. The glass jars were filled in a sterile environment inside a vertical laminar-flow hood (AHC–6D, ESCO, Horsham, PA, USA). A total of sixty jars were filled. Twenty-five jars were stored at a room temperature of 30 ± 2 °C and exposed to luminous stress (5435 Klux), and thirty-five jars were wrapped with aluminum foil and refrigerated at 5 ± 2 °C. The light stress was carried out under a special chamber of white fluorescent light emitters with a full light spectrum. This type of light was selected, because it is the most common presented in the markets.

### 3.6. Sample Preparation for Alkaline Hydrolysis

Prior to the alkaline hydrolysis analysis, the PIBP sample was shaken in an ULTRA-TURRAX at 35,000 rpm for 1 min. For the preparation of the sample, the method of [52] was followed with few modifications. PIBP (3.0 g) was mixed with 2 mL of *n*-hexane, 2 mL of dimethyl sulfoxide, 3 mL of acetone (all reagents of HPLC grade) and 1 mL of brine solution in 15 mL glass conical centrifuge tubes. The sample was shaken with a vortex model VM–300 (Gemmy Industrial Corp., Taipei, Taiwan) for 1 min, and the tubes were centrifuged at 3500 rpm for 3 min. The *n*-hexane layer containing astaxanthin was transferred with a pipette into a clean tube containing 1 g of Na_2_SO_4_. The *n*-hexane layer was then transferred into a 50 mL flask, and the solvent was evaporated to dryness at 50 °C with a rota-evaporator model RE-52a (Zhengji, China). It was then redissolved in 3 mL of acetone, adding 1 mL at a time, by mixing in an ultrasound bath model UC–30a (Biobase, Jinan, China). The acetone extract was used to adjust the absorbance within 0.8 and 1.2 (analysis performed in triplicate). Of the sample, 3 mL was used in the alkaline hydrolysis.

### 3.7. Alkaline Hydrolysis

The sample with acetone was dried with nitrogen gas flow, and the content was dissolved in 2 mL of methanol. The alkaline hydrolysis was carried out according to the method described by [52]. A solution of 1% of KOH (0.1 mL) was added and mixed in the vortex. The astaxanthin esters were hydrolyzed at room temperature under the influence of nitrogen gas in the dark for 18 h. The methanol phase was extracted with petroleum ether, and the organic phase was washed with brine solution. The petroleum ether was placed in a clean tube with Na_2_SO_4_, and the solvent was removed by nitrogen gas flow. These were redissolved in 3 mL of acetone and analyzed by HPLC.

### 3.8. HPLC Analysis

The HPLC was conducted on a liquid chromatograph model 7100 (Hitachi, Japan) equipped with three pumps and a UV-Vis detector. An aliquot of 20 µL was taken from the sample using an RP-18 column. The mobile phase consisted of a mixture of solvents: A (acetone), B (methanol) and C (water). For this analysis, the elution procedure used was 60:23:17 A:B:C (*v*/*v*/*v*) for 2 min; a linear gradient of 60:30:10 A:B:C (*v*/*v*/*v*) was applied for 4 min. The mobile phase was pumped at a flow rate of 1 mL/min and was detected at a wavelength of 474 nm. The astaxanthin identification was carried out by comparing the retention time with the astaxanthin reference standards injected in the HPLC system for the production of the astaxanthin standard curve. These reference standards were prepared using standard solutions (1 to 80 ppm) of astaxanthin. The concentration of astaxanthin in the samples was calculated from the standard curve.

### 3.9. Control Variables

For all assays, 25 mL of PIBP was transferred into a 50 mL beaker and mixed with an ULTRA-TURRAX at 35,000 rpm for 1 min. A digital refractometer model PAL–3 (Atago, Tokyo, Japan) was used to determine the soluble solids; the results were expressed in °Bx. To determine the pH index, the pH meter electrode (Mi–151, Martini Instruments, Rocky Mount, NC, USA) (calibrated with buffer solutions pH = 4.0 and pH = 7.0) was used. For the determination of chlorides, one drop of the PIBP sample was added to the salinity digital refractometer (model HI96821, HANNA, Woonsocket, RI, USA), and the results were expressed in grams of NaCl/100 mL. The two refractometers were calibrated prior to their use in the experiments.

### 3.10. Oxygen Radical Absorbance Capacity (ORAC) Assay

The ORAC method is widely used for assessing antioxidant capacity in biological samples and food [53]. The assay consists of measuring the decreased fluorescence of a protein as a result of loss of its conformation when it suffers oxidative damage caused by a source of peroxyl radicals; hence, the method is capable of measuring the antioxidant ability of the sample to protect the protein (fluorescein) from oxidative damage [54]. Currently, the assay is able to measure the hydrophilic and lipophilic antioxidants through the use of different solvents with different polarities as acetone/water/acid acetic in a gradient [55].

ORAC assays were performed for samples of AOE. ORAC values were determined using the method described by [56], which in turn is based on the method of [57] with a few modifications. The area under the fluorescence decomposition curve (AUC) was used in the calculations through a linear regression equation in trolox standards. The net area under the curve was obtained by subtracting the area below the curve for the blank values of the sample curves and standards. Finally, the ORAC value was expressed in µmol of trolox equivalents per gram (µmol of TE/g).

### 3.11. Determination of Total Polyphenols Content

Total polyphenols content was performed for samples of AOE following the Folin-Ciocalteau method [58]. Gallic acid calibration solutions were made to obtain a standard curve. The total polyphenol of samples were performed on the acetone/water/acid acetic extracts and were calculated on the basis of the standard curve for gallic acid. The results were expressed as milligrams of gallic acid equivalents per gram (mg of GAE/g).

### 3.12. Statistical Analysis of the Results

The experimental results were expressed as mean values and their standard deviations. The statistical evaluation of results was carried out using the spreadsheets of Microsoft Office Excel 2010 software. The comparisons of the samples were performed using the unidirectional analysis of variance (ANOVA), and when differences were observed, Duncan’s multiple range test was applied. The differences were considered significant when *p* < 0.05.

## 4. Conclusions

The enrichment of beverages with carotenoids may reduce the incidence of certain diseases. However, the use of carotenoids in foods is currently limited because of their poor water-solubility, high melting point, low bioavailability and chemical instability. Commercial isotonic beverages with added synthetic pigments obtained values of $a^{*}$ and $b^{*}$ that places them in the first quadrant of the sphere corresponding to the color space CIE $L^{*}a^{*}b^{*}$ without major differences between them in terms of luminosity $L^{*}$. The emulsion prepared from astaxanthin oleoresin (AOE) can be classified as a “macro-emulsions” because of the area of micelles oscillating between 0.15 and 7.60 µm^2^.

The stability study carried out for samples after three months of storage at T = 5 ± 2 °C and in dark conditions confirmed the solubility of AOE in the commercial colorless IB—this added a red-orange color that simulates the red-color commercial IB. The PIBP, under intense luminosity, showed greater color instability because of an accelerated degradation under this stress, thus leading to the total loss of color within a period of eight days. PIBP stored in darkness presented higher stability, because it maintained its concentration during the three months at T = 5 ± 2 °C. Despite this, after the initial seven days, the concentration of astaxanthin was decreased by 25% of its initial value.

For future research, studies in parallel with the commercial and researched beverages will be considered.

## Figures and Tables

**Table 1:** Chromatic coordinates obtained from the different commercial isotonic beverages present in the market (*n* = 30).

Commercial Isotonic Beverage	Brand of Beverage	Chromatic Coordinates			$\frac{a^{*}}{b^{*}}$
		$L^{*}$	$a^{*}$	$b^{*}$	
Colorless	A	61.72 ± 0.56	−0.98 ± 0.13	1.74 ± 0.17	−0.56
Orange	A	47.43 ± 3.16 ^a^	24.00 ± 0.86 ^a^	78.21 ± 2.21 ^a^	0.31
	B	46.57 ± 0.34 ^a^	22.58 ± 0.17 ^b^	75.41 ± 0.67 ^a^	0.30
Red	A	33.34 ± 0.17 ^b^	45.09 ± 0.06 ^b^	44.40 ± 0.45 ^b^	1.02
	B	35.74 ± 1.32 ^a^	41.96 ± 0.34 ^a^	29.69 ± 2.17 ^a^	1.41

X ± S: mean value ± standard deviation. Different letters in the column values for each coloration of beverages indicate statically significant differences for *p* < 0.05.

**Table 2:** Chromatic coordinates obtained from colorless isotonic beverages with an addition of astaxanthin oleoresin emulsion (AOE) (*n* = 10).

Pigmented Isotonic Beverage	Concentration of AOE (ppm)	Chromatic Coordinates		
		$L^{*}$	$a{\;}^{*}$	$b{\;}^{*}$
1	530	42.36 ± 0.06 ^a^	28.59 ± 0.04 ^a^	39.54 ± 0.21 ^a^
2	992	35.07 ± 0.03 ^b^	35.49 ± 0.04 ^b^	48.56 ± 0.20 ^e^
3	1548	30.74 ± 0.05 ^c^	38.00 ± 0.06 ^c^	47.19 ± 0.16 ^d^
4	2076	28.18 ± 0.04 ^d^	38.80 ± 0.05 ^d^	44.31 ± 0.20 ^c^
5	2520	25.80 ± 0.03 ^e^	39.20 ± 0.06 ^e^	41.09 ± 0.17 ^b^

X ± S: mean value ± standard deviation. Different letters in the column values indicate statically significant differences for *p* < 0.05.

**Table 3:** Color differences (∆E) between red-color and orange-color commercial isotonic beverages (IB) with prototype pigmented isotonic beverages (PIBP) with different concentrations of astaxanthin oleoresin emulsions (*n* = 10).

Formulas	Concentration of	∆E Between Commercial IB and Developed IB			
		Brand (Orange-Colored)		Brand (Red-Colored)	
Pigmented IB	AOE (ppm)	A	B	A	B
1	530	39.27 ± 0.18 ^c,a^	36.61 ± 0.20 ^c,b^	19.42 ± 0.11 ^a,a^	17.88 ± 0.14 ^c,b^
2	992	34.11 ± 0.17 ^e,a^	31.94 ± 0.22 ^e,b^	10.60 ± 0.08 ^b,a^	19.96 ± 0.19 ^a,b^
3	1548	37.90 ± 0.18 ^d,a^	35.84 ± 0.17 ^d,b^	8.05 ± 0.09 ^d,a^	18.63 ± 0.15 ^b,b^
4	2076	41.70 ± 0.22 ^b,a^	39.61 ± 0.15 ^b,b^	8.14 ± 0.03 ^d,a^	16.76 ± 0.19 ^d,b^
5	2520	45.57 ± 0.15 ^a,a^	43.43 ± 0.18 ^a,b^	10.13 ± 0.07 ^c,a^	15.38 ± 0.15 ^e,b^

The first letters in the exponent refer to the comparison of the mean values in the columns and the second letters to the comparison of the mean values in the rows at a confidence level of *p* < 0.05.

**Table 4:** Obtained values in some control variables of the prototype isotonic beverage pigmented (PIBP) stored in darkness and under light stress.

Control Variables	Days	PIBP	
		Darkness	Under Light Stress
Soluble solids (°Bx)	0–30	1.3 ± 0.20 ^a^	1.3 ± 0.20
	31–90	1.4 ± 0.20 ^a^	------
pH	0–14	4.07 ± 0.02 ^a^	4.15 ± 0.01
	15–35	4.09 ± 0.02 ^ab^	------
	36–90	4.13 ± 0.02 ^b^	------

X ± S: mean value ± standard deviation. Different letters in the same column indicate a statistical difference for *p* < 0.05.

**Table 5:** Comparison of total phenols content (TPC) and oxygen radical absorbance capacity (ORAC) of some standard food and functional foods. GAE: gallic acid equivalents and TE: trolox equivalents.

Products	TPC (mg GAE/g)	ORAC (µmol TE/g)	Ratio ORAC/TPC
Sweetener	0.02 ± 0.003 *	0.08 ± 0.02 *	4.00 **
Sweetener with Green tea extract	1.43 ± 0.11 *	25.56 ± 1.9 *	17.87 **
Yogurt drink	0.16 ± 0.02 *	1.76 ± 0.25 *	11.00 **
Yogurt drink with catechins	0.41 ± 0.05 *	8.99 ± 1.06 *	21.93 **
Isotonic beverage	0.18 ± 0.05 *	1.13 ± 0.04 *	6.28 **
Isotonic beverage with green tea extract	0.63 ± 0.08 *	5.07 ± 0.20 *	8.05 **
Isotonic beverage with AOE*	0.008 **	0.24 **	30 **

* Values obtained in [45]. ** Values obtained in this paper.
